# Notes on Belgian Lunatic Asylums, Including the Insane Colony of Gheel

**Published:** 1857-04-01

**Authors:** John Webster


### Art. II.—NOTES ON BELGIAN LUNATIC ASYLUMS, INCLUDING THE INSANE COLONY OF GHEEL.

By John Webster, M.D., F.R.S., &c.

(*Continued from page* 78.)

### Gheel.

Amongst the most remarkable localities of Europe, in reference to its inhabitants, none seems so singular, stands more prominently forward in various phases, or deserves inspection by philanthropists and curious travellers, like the establishment for lunatics, which forms the subject of subsequent observations. Situated in the north-eastern portion of Belgium, towards the confines of Limburg, and formerly occupied by the Texandrians, mentioned in Cæsar's *Commentaries*, but subsequently called the Siberia of the Low Countries, Gheel long remained almost unknown to foreigners. Until very lately, no roads existed through this outlandish district; consequently, it was rarely approached, even by the natives of neighbouring provinces. Nay, in 1821—when Esquirol paid it a visit, with my friend M. Voisin—the journey then proved a most difficult undertaking. Indeed, so recently as the Revolution of 1830, only one crazy lumbering vehicle, of the rudest description, carried travellers twice a week from Lierre—a cantonal town of 13,000 inhabitants—which is situated at the confluence of the great and little Nethe rivers, about eighteen miles towards Malines. This distance, however short, often required from nine to ten hours travelling; the carriage being frequently up to its axle in mud or gravel, according as weary wayfarers then experienced wet or dry weather.

At present matters are entirely changed; and those who undertake the same journey, which only twenty years ago was surrounded with many difficulties, need not now hesitate, notwithstanding former discouraging statements. These accounts belong to history, of which a parallel no longer remains; as the following outline of my recent journey to Gheel amply demonstrates. Leaving Brussels early by railway, I stopped at Contich, the second station south of Antwerp. From thence a train immediately started for Hérenthals, on the Turnhout line, where it arrived about an hour afterwards. There, a two-horse omnibus was waiting, to carry passengers to Gheel, eight miles further, in which town I arrived in about one hour and a half, without encountering mishaps, or experiencing the slightest fatigue; having thus travelled the whole distance in little more than four hours, but altogether very differently from that described by former tourists visiting Campine.

For the information of those who may hereafter propose to sojourn at Gheel, I would briefly mention that two good hotels are open to receive strangers. Both may be entered with confidence; but the one I selected was "De Schild Van Turnhout," kept by M. Fr. Wouters. Certainly, it has seldom been my good fortune, while travelling, to obtain better lodgings, and experience more satisfactory treatment, in all respects, than at this hostelry. The bed was excellent, linen white as driven snow, with unexceptionable food, and all cooked admirably. Doubtless, the sanded rooms lacked carpets, and the attendants were not stiff neckclothed waiters, like those often seen at English hotels, while serving in silver dishes burnt mutton-chops or tough beef-steaks; which frequently cause fits of indigestion, not alleviated by an expensive bill afterwards presented. There, on the contrary, a smiling servant-girl attended at table who, although unable to speak either French or German, and only her own native Flemish tongue, yet understood whatever the guests required. To these details I would also add, that the cost was extremely moderate. Thus for breakfast, consisting of better *café au lait* than one often gets in England, with unexceptionable bread-and-butter *ad libitum*, the charge was sixty-five centimes; or only six pence halfpenny sterling! For dinner, composed of soup, three kinds of excellently cooked viands, vegetables, followed by cheese and dessert, I paid one franc and a half; while another franc per night was added for the sleeping apartment. In short, nothing could be more satisfactory in every respect. Therefore, should future Gheelois pilgrims propose making a journey thither, they need not apprehend suffering perils or privations; but quite the reverse. My sojourn and treatment at the "Turnhout Arms" hence proved extremely agreeable; and when returning to Hérenthals, the inside of our conveyance was filled with twelve young ladies, going home from a school of repute near Gheel, to pass their autumnal holidays at Antwerp and Brussels. These facts are now mentioned to indicate, that this district, formerly considered almost beyond the pale of civilization, and but little known even by residents of adjacent provinces, has now joined in the onward march of improvement.

### THE INSANE COLONY.

According to tradition, the town previously named, and capital of Campine, has for ages been celebrated as a refuge for lunatics; in which, since long bygone times, it was popularly believed, a residence would prove greatly conducive towards an insane patient's recovery. The legend respecting the origin of this reputation is thus reported:—Late in the sixth century,

Dympna, a daughter of an Irish king, became converted to Christianity, by an anchorite named Gerebert. The father felt much enraged at this conversion, and being also amorous, it is said, of his own child, threatened vengeance; upon which the young lady, with her male companion, fled across the sea, and arrived safely at Gheel; where, she resolved to dedicate herself to devotion and celibacy, along with St. Gerebert. However, the old pagan sovereign having discovered their retreat, insisted upon Dympna again changing her religion; but she would in no way consent. This refusal made the savage monarch so furious, that he drew his sword and cut off her head mercilessly at one blow; having done the same previously to Gerebert. These cruel acts greatly frightened several lunatics, said to be then present; and tradition reports cured them immediately, through the strong impression this horrible spectacle produced on their excited feelings. Immediately, the cry of "A miracle! a miracle!" was raised by the wondering bystanders; and thus Dympna—saint and virgin—became ever after the patron of all mad persons. This faith having spread abroad, lunatics were brought to Gheel to get cured through St. Dympna's intercession, and firmly established its reputation. About A.D. 1200, a church was erected on the spot, where the murder above described had been committed, in which the saint's bones and relics were subsequently deposited.

The principal altar of this ancient sacred edifice—which deserves inspection—represents an allegorical figure, larger than life, of St. Dympna—not headless—seen elevated on a cloud imploring the Divine mercy upon several lunatics standing in her vicinity. At each side, groups of insane persons—all large statues—may be likewise observed, whose feet and hands are bound by golden chains, identical in form with those still used to restrain violent maniacs. In a central chapel of the "diambulatorium," an elaborate carving in oak—much admired by sculptors—represents the tragical history of this church's patron saint. In the first compartment of that curious work—made by an unknown artist—the birth of Dympna is portrayed. The second typifies the Queen her mother's death. In the third, the devil appears tempting the Irish sovereign. The fourth shows Dympna embarking on board of ship with St. Gerebert, in order to cross the ocean. The fifth exhibits the King in pursuit. In the sixth, he is seen cutting off his daughter's head, close to the decapitated corpse of her pious instructor. In the seventh, several priests—wearing rich habiliments—are carrying the saint's relics in a grand procession. Finally, in the eighth compartment, the devil is observed issuing from the head of a female lunatic, while prayers are being said by some priests; and near whom another chained maniac seems anxiously waiting his turn of deliverance from the demon by whom he is supposed to be possessed.

But the most remarkable object, perhaps, in this church, is a highly ornamented tomb, said to contain the saint's bones. This tabernacle stands on four stone pillars behind the great altar, so as thus to form a passage about three feet in height, and through which lunatics, brought here for cure, were accustomed to pass on their knees. Poets say the palace stairs of great personages are often worn by beggars asking favours. Here, the poetical sarcasm is really verified; since, the stone floor of this formerly much-revered locality appears worn away, to some extent, by the pressing limbs of devotees; analogous, in fact, to the marked result I observed on the bronze figure of St. Peter, at Rome, whose toe—not the original appendage, as a joining near its ball shows the present has been added—was actually much decreased in size by continuous osculades from faithful "pellegrini," frequenting the Eternal City's magnificent cathedral. This phenomenon I personally noticed, when examining the above celebrated statue, which is believed to have been cast from the metal forming, or as some authorities confidently assert, once was the real "Jupiter tonans" of antiquity.

At Gheel, these idolatrous genuflexions, under St. Dympna's tomb are now of much rarer occurrence than in more ancient times, so that cures through such means have become, of late, very unfrequent: or, to quote the words of a late writer, "ever since faith has been extinguished and religion almost exiled from the earth." Nevertheless, examples still sometimes occur, where persons devoutly crawl on bended knees through this once-hallowed spot; as well to get cured, as to prevent their subsequently being attacked by mental aberration. Indeed, this ceremony actually took place not long previous to my visit; the party and assistants, all the while, then singing or praying, in order thus to obtain the saint's intercession in the lunatic's favour; whereby they believed, the ultimate result would prove more efficient and satisfactory.

Near the central part of this temple, on the left of the choir, St. Dympna's statue, clothed in silken garments, and profusely ornamented with gold lace, fills a large glass case, before which wax tapers and a "*prie-Dieu*" are placed, ready for any person to use, who might desire to address the image in question. This figure once possessed great repute; and although less now than formerly, it was reported to me that demented persons still frequently visit the locality mentioned, but always accompanied on such occasions by some attendant; since an express regulation forbids any lunatic frequenting the churches at Gheel, without a sane companion.

In addition to the peculiar features above described, characterizing this far-famed sacred edifice, the most singular place of all is, indubitably, a darkish dungeon-looking apartment, in a small house attached to the principal church tower, now occupied by two female officials. Within this hole—apparently a kitchen—maniacs brought for cure were lodged by their relatives, for the space of *nine* days consecutively. During the whole of that period, the insane victim remained chained close to the fireplace, by iron rings fixed on one wrist, having another on the ankle; both being still seen as originally arranged. At night, the lunatic was put into a wooden bed adjoining the chimney, to which strong iron chains bound its occupant by the feet, as also both arms, having straw under, and some covering thrown over the wretched sufferer. I carefully examined the fetters of this real Procrustean couch—all being the original implements—and thought the links sufficiently strong and massive to restrain any prisoner, however violent; nay, by bulls or tigers they could not have been readily broken.

On one side of this room, but close under the roof, a confined gallery exists, from whence relatives, or favoured curious spectators, could witness whatever mystical ceremonies might be performing below. Throughout the entire nine days—always considered essential towards ensuring recovery—*nine young virgins*, hired for that purpose, made a daily procession round the church aisles, passing *nine times* on their bended knees under St. Dympna's tomb; all the time invocations being offered up for the chained maniac's recovery, at the same period that certain formulæ were gone through in the patient's presence; whilst a priest likewise recited prayers appointed for such occasions, besides imparting to his afflicted auditor religious consolation.

Formerly, proceedings like those detailed were much more common, than in the present enlightened age; and these brought, sometimes, a considerable revenue to the various performers. The maniac's board had to be paid to the owners of so famed an apartment, wherein the party was lodged. The priests received their "honoraria," and the *nine* young ladies did not chant, walk in procession, or even creep under St. Dympna's relics gratuitously; but quite the reverse. Nay, if the patient could not attend personally, a substitute had to be hired to make the ceremony full and complete; the rule here being, as elsewhere, "*Point d'argent, pas de Suisse.*" Hence, the expense of such performances proved often no trifle, but even of some amount. Although the superstitious practices just narrated appear almost fallen into desuetude; nevertheless, examples have occasionally taken place during very modern times, of which an instance was related, with all its details, which happened recently. But whether the poor maniac for whose benefit the ceremony was then performed got effectually cured, my reporter could give little satisfactory information.

Sometimes, it was stated, persons have been brought hither, alleged to be insane from interested motives; and, if not really mad before, thus to try and make them so by cruel treatment, as the following authentic illustration, communicated by a medical friend of mine, who was consulted professionally, amply testifies. The father-in-law of an heiress much wished that young lady to marry his own brother, and thereby retain her fortune in the family. However, her heart being already in the keeping of a more juvenile and longer known suitor, she would not agree to any such money arrangement. This refusal enraged the proposers; and therefore a rumour was industriously spread abroad, that their intended victim had become crazed. To verify the fact more publicly, Miss —— was in due time transferred to St. Dympna's chamber; where, all the requisite formalities were followed, but without the desired result. Afterwards, the stepfather tried to obtain a certificate of insanity from Dr. ——, in order that she might be consigned to an asylum; but the physician refused to grant any such document, as he could perceive no decided evidence of mental alienation. Fortunately, during several professional interviews with his patient, Dr. —— heard the whole story, and how harshly this love-sick maiden had been treated. Whereupon, he took the proper steps to get her liberated from all control of either parent; and proving successful, she now lives elsewhere unmolested, the selfish brothers being thus completely baulked of their greatly-coveted prize.

Notwithstanding the present sceptical world entertains much less faith respecting the sanative powers of St. Dympna to cure mental diseases than formerly, but which prevalent incredulity fanatics ascribe to the wickedness of mankind; still, whenever the saint's annual feast-day occurs, viz., about Pentecost, thousands of people, from many neighbouring or even distant villages and towns, flock into Gheel, to behold the ceremonies which then take place, mix with the crowd, and otherwise enjoy themselves. During *nine days*, the saint's relics, with her shrine also, remain exposed in the church to public gaze; whereby, the assemblage of sight-seekers and idlers often becomes very great, whilst many devotees may even be seen crawling through the narrow passage under the hallowed tomb. After performing this act of blind devotion, these parties hope they will never afterwards become insane, through St. Dympna's intercession. Whatever sensible people may think of similar proceedings, and of their saturnalian character, a large expenditure is thereby caused in the town; much to the advantage of shopkeepers, cabaret hosts, and the ultimate pecuniary profit of numerous inhabitants. On the last occasion when this fête was celebrated—only a few months past—such an immense concourse of visitors then arrived, that Gheel was quite full for an entire week, and the streets seemed constantly crowded; all the time, most people appearing chiefly bent upon amusement, pleasure, and physical gratification.

Various authors might be named respecting the early history of Gheel, the discovery of St. Dympna's remains, and the great reputation which this locality has long enjoyed, as a favourite refuge for lunatics; but one or two quotations will suffice. Thus, M. Gife states, in a work published by the Literary Society of Turnhout, that, about the seventh century, a chapel was built in honour of St. Martin, around which nearly 700 houses were soon erected. Again, M. Gazet says, in the Ecclesiastical History of the Low Countries, dated 1614, that, led by popular tradition and belief, the clergy searched for the bones of St. Dympna and St. Gerebert, which they actually discovered—as he asserts—buried in two stone coffins, white as snow, although only dark-coloured rocks were found elsewhere. This peculiarly strange circumstance was therefore believed to have been the work of angels, to indicate these holy martyrs' candour and chastity. Lastly, in a volume published in 1658 by M. Craywinkel, attached to the Abbey of Tongerloo, it is gravely asseverated, "if the wonderful cures witnessed of insane persons resident at Gheel were not real miracles, at least they must be held as astonishing recoveries, effected wholly through the powerful intercession of the holy virgin, who has become patron saint of this locality;"—these facts being, he then adds, extracted from an ancient yet authentic register, belonging to the collegiate church of Gheel, and consequently implying that no mistake could prevail regarding such narratives!

The locality where insane patients have, during many centuries, been associated with the general population, constitutes part of the province of Campine, or "*Kempen-land*," which means, flat and without trees; whereof the capital is Gheel. The district now particularly referred to forms a level rather elevated plain, about three leagues in diameter, having a fertile soil, but surrounded, on several sides, by open sandy fields, or steppes of considerable extent; while turf being in many places dug as fuel, these parts hence afterwards seem very like stagnant marshes. The immediate environs are much more productive than adjacent districts, which still continue often covered with broom or stunted trees; whereby, this central division of the commune truly resembles an oasis in the desert. Again, the town itself occupies a moderate elevation, lying betwixt the river named the Great Nèthe, and the two smaller streams called the eastern Nèthes; but, as winter often proves severe from strong northeast winds, and snow or frost, followed sometimes by hot summers, while damp weather is not uncommon, this portion of Belgium is not considered salubrious. Another drawback should likewise be enumerated; viz.,—the water used by residents is so much charged with sulphate of lime, that it virtually becomes improper for domestic purposes. However, most excellent water could be easily brought by pipes from the sandy hills near Casterlé, towards Turnhout; which possesses such superior qualities, that parties have proposed to make a reservoir at Casterlé for the supply of Brussels.

Influenced by the causes just enumerated, intermittent fevers, typhus, and pectoral diseases in winter are by no means unfrequent; such results arising or being greatly promoted through the cold soil and humid habitations, occupied especially by peasants. Nevertheless, Gheel is considered a more healthy situation than the adjacent villages. It seems also rather pretty for a country town, has some good, and one long broad street; while the central square or "Place" is rather large, contains mostly two-storied houses, and several well-furnished shops; while the cathedral-church of St. Amand occupies one end of this enclosure. The urban population amounts to nearly 4,000 persons; that of the whole commune being at least 9000, of whom, on an average, there are always nearly 800 certified lunatics; thus giving one insane to every twelve sane residents.

Before proceeding to describe the chief features characterizing the lunatic population of Gheel, their general treatment and superintendence, some outline of the chief regulations specially affecting this singular colony will be neither inappropriate nor uninteresting. Besides the laws applicable to every insane asylum throughout Belgium, and being also placed under the inspection of the permanent commission charged with the special surveillance of lunatics, an additional code, or new "Règlement," dated 31st December, 1852, was promulgated by the Minister of Justice for its government, but afterwards modified by a late Royal Decree. According to these laws the colony of Gheel is now regulated, the chief authority being vested in a committee of eight members, who superintend the entire establishment. Of the above, four are annually appointed by the superior Commission, either from their own body, or of persons resident in the commune of Gheel, and places adjacent. To the parties thus nominated, the Minister of Justice adds four other members, taken from a double list of candidates submitted for his choice, by the communal council. Over this managing committee so selected, the Burgomaster of Gheel presides, or one of the sheriffs, who then has a deliberative voice. These officials are especially charged with the receipt of all moneys received as board for lunatics; they pay disbursements, distribute the patients in different dwellings, besides watching over their interests and treatment. Further, the committee likewise see that the hosts and hostesses perform the duties required, towards any inmate consigned to their keeping. Every month, one member of the surveillance committee is nominated as visitor, whose delegated function is to preside when patients are admitted and discharged; but no admission or dismissal of any inmate can take place without previously consulting the inspecting physician.

All householders, within the commune, authorised to receive lunatic inmates as boarders, are divided into two classes, one being denominated "hosts," the other foster-fathers or "nourriciers;"—the former being those persons licensed to take insane patients paying at least 25 francs per annum more than indigent lunatics; the latter, or "nourriciers," comprising individuals who receive inmates at the minimum rate of payment. Both these parties are inscribed on a register kept on purpose; the distribution of patients newly arrived being made, as much as possible, according to rotation, still leaving relatives power to select any registered person with whom they would prefer placing the lunatic.

Unless under very particular circumstances, not more than three lunatics can reside in one dwelling-house, while two or more insane boarders are not allowed to occupy the same bedchamber. Special sanction may, however, be granted by the permanent committee to admit a larger number, after they have received a report thereon from the divisional and inspecting physicians. When such exceptional permissions are given, the room occupied must always contain fifty cubic feet of air to each resident; the roof and walls being lime-washed, twice a year at least. Strict rules are also laid down respecting the furniture, the food supplied to inmates, and their clothing, which must consist of woollen stuffs in winter, with cotton in summer; but without distinctive patterns, or made like an uniform. Again, their body-linen should be changed always once weekly, or oftener when necessary, especially dirty patients. Lastly, no lunatic can be employed in any bodily labour, unless considered judicious by the sectional physician, and provided it does not induce injurious fatigue.

During summer, insane residents are not allowed to leave their domicile prior to six o'clock in the morning, or remain longer from home than eight in the evening; the hours being 8 A.M. to 4 P.M. in winter. They are not permitted to enter any cabaret, excepting tranquil patients who may require refreshment; but it is then expressly prohibited to serve even these with spirituous liquors, and none can smoke near a stack-yard with uncovered tobacco-pipes. Other stringent police regulations regarding lunatics are in force, which it seems superfluous to mention specially. However, if any inmate escapes, the party with whom the individual lodged, besides losing all remuneration for those days the fugitive is absent, must pay three-fourths of the expenses incurred by recapture; the guardian of the section to which such patient belonged being mulcted the other fourth. Each lunatic has a book, or "livret," as it is called, in which, besides the name with various other particulars, all clothes supplied, payments made, remarks of sectional or inspecting physician, and observations by official visitors, are entered; so that, from this record, whatever is important or necessary to be known respecting any particular patient can thus be easily ascertained.

The entire commune being now divided into four sections, separately comprising a certain defined district, the total insane residents hence form as many distinct divisions; each having a head guardian, and one physician, to whom is committed the special medical care of every inmate belonging to that section. There is one consulting surgeon for the entire district; whilst over all is placed an inspecting physician, who of course possesses the chief authority in everything connected with medical treatment, and professional superintendence of the whole establishment. Four pharmaciens are likewise appointed, one of whom supplies any medicines ordered by the physicians or surgeon; each for three months alternately. When I visited Gheel last September, the medical staff was constituted as follows:—viz., Dr. Bulkins, the inspecting physician; Drs. Van Vitzen, Bœckmans, De Backer, and Verbist, sectional physicians; with M. Giroltt as consulting surgeon; all well qualified officers for their respective appointments.

With reference to the duties of the inspecting visitor, it may be observed, he can enter at any time he pleases, and without previous notice, every dwelling within the commune. He may cause a patient to be produced, examine the room occupied, the bed, and clothing; also hear all complaints, or make whatever special inquiries he deems necessary; infirm, dirty, epileptic, paralytic, and helpless lunatics being considered the most worthy objects of attention. The visitor may also order the removal of any patient to another abode, should sufficient cause appear; or when neglect, cruelty, and improper treatment has been practised. Lastly, wherever a nourricier becomes convicted of having struck any lunatic inmate, unless it be conclusively proved that such act was solely and legitimately in personal defence, the offender is then stigmatized as *infamous;* and hence incapacitated afterwards to receive patients, until such ban is removed.

When I inspected Gheel, the total insane population under treatment comprized 774 persons, as previously stated. Of these, 409 were male and 365 female lunatics; the town, or first section, being then most numerous. One-half of the whole number were classified as capable of, and indeed actually employed in bodily labour, a large majority of whom comprised females; while the remainder were reported as idle or unable to work, the greatest number of that category belonging to the male sex. Amongst the total inmates, the largest proportion, or 350 cases, were examples of mania and its varieties. Dementia came next, of which were found 265 instances. Melancholia showed 60 cases. Epilepsy supplied 51 instances, the males being 25, with 26 females; whilst the remainder exhibited less marked varieties of mental disease. The 774 patients now enumerated were distributed in nearly *five hundred* different dwellings; whereof about 300 were cottages or farm-houses in the country, the rest being residences in the town of Gheel.

With regard to the various payments received for patients differently classed, it may be mentioned that the first division—comprising 49 lunatics—paid from 400 to 1200 francs annually, according to the accommodation supplied; the second—amounting to 147 cases—paid 300 francs; the third—including 266 individuals—275 francs, or 75 centimes per day; while all the rest, or fourth class—being 312 persons—were maintained by their nourriciers at the small sum of 238 francs per annum. These respective amounts included everything. But, however cheap food and lodging may still prove in "Kempen-land," the actual profit must be almost *nil*, out of nine pounds ten shillings sterling; after feeding, clothing, and lodging an adult lunatic boarder. Undoubtedly, in many instances, their labour is given besides, but not invariably; and, although industrious working inmates are more in request than idle patients, parties must take their chance, with reference to such contingencies.

Accompanied by a head attendant, and the inspecting physician—Dr. Bulkins, who most kindly showed me everything worth noticing—I visited numerous houses in the town, and a great many cottages scattered over the adjoining country; in which, often one, although most frequently two, and occasionally three insane persons resided. That is the general system followed, with but very few exceptions; seeing, not more than five instances exist throughout the entire colony, where beyond four patients are placed with the same family, but only then for special reasons, and after an express authorization from the committee of inspection is obtained. Being occupied during two consecutive days in visiting various abodes, ample opportunities were thus afforded for seeing whatever was either interesting, important, or peculiar; and, consequently, thus form some general opinion respecting the establishment. With reference to patients residing in rural dwellings, it should first be remarked that, amongst the seventeen hamlets of this commune—some being even like villages—in three no lunatics are received; and, however anxiously most householders often desire to obtain insane boarders, throughout all the neighbouring communes an opposite feeling is very prevalent.

Speaking generally, the sexes are usually lodged in separate houses. However, in regard to very aged persons, whose disease was chronic, and were of quiet inoffensive dispositions, a man occasionally lived in the same family where an insane old woman also resided. All suicidal, dangerous, homicidal, or mischievous insane patients are rarely if ever received as inmates at Gheel; while, on the other hand, should any resident subsequently come under these categories, they are forthwith sent home, or to an asylum elsewhere. Again, the authorities generally place boisterous, idle, or agitated maniacs in remotely situated and solitary cottages or farm-houses, which occupy the open heaths; where, such lunatics, having no neighbours, cannot disturb any person, or cause much annoyance. These, although excited or very noisy, may there walk about without danger to others or themselves; being thus placed beyond the observation of bystanders, not near similarly afflicted fellow-creatures.

When perambulating the various hamlets visited—often through pretty but devious pathways—we frequently noticed lunatics occupied as agricultural labourers in adjoining fields; whilst some were quietly walking to or from neighbouring cottages, quite as tranquilly as ourselves. Being all well acquainted with the inspecting physician, these parties saluted him respectfully, and often conversed with us familiarly; in short, they behaved like ordinary peasants, or any rational person. We saw others sitting at the cottage doors, and some looking out of windows; while several were amusing themselves with children of the family, in adjoining gardens or enclosures. In one of the public roads we met a maniac, who lived in a cottage at some distance, then carrying an infant in his arms like any nurse. He seemed to take great care of his innocent charge; and the physician remarked that such an occupation constituted this lunatic's chief enjoyment. Afterwards, we encountered another insane resident—a young man—amusing himself with three little children, romping with them, and at the same time taking care they did no harm. In a solitary lane, we next came up with a patient, who was being conducted homewards to dinner by the juvenile daughter of his host, after labouring in an adjacent field. In various other instances, we observed insane persons sauntering about; and some also going towards or returning from neighbouring farm-houses. In truth, had it not been from the vacant-looking countenances noticed in most cases, and occasionally that their legs were loosely tied together by leathern thongs—so as to prevent the wearers from running fast, or going to any distance—I should scarcely have recognised many of the parties then enjoying themselves, while breathing the pure and open air of heaven, as real lunatics residing in the Gheelois commune.

Within the houses and cottages we inspected, many insane residents were occupied as ordinary servants; some superintending cows, churning, labouring in the barn, cooking food, cleaning the house, rocking the cradle, and taking care of the children; in short, employed much in the same way as they might have been at home, or out at service. Various male patients, again, were labouring in the gardens or fields; others working in carpenters' shops; also smithies, stable and farm yards; besides being engaged in such out-door employments as are common amongst any agricultural population;—these occupations having this very great advantage for lunatics, that whilst undergoing physical exertion—often so beneficial for their mental malady—they, at the same time, are much in the open air, breathe a purer and more salubrious atmosphere than most inmates of wards, day-rooms, or frequently too-confined work-shops, can enjoy in modern asylums. In several houses, we also observed female lunatics comfortably sitting with their hostess at table knitting, sewing, making clothes, and conversing as if equals, friends on a visit, or relatives. Such spectacles were truly pleasing; and when looking out of the cottage-windows near such parties, upon sometimes a pretty flower-garden, or towards open green fields, strangers could hence scarcely suppose, from outward appearances and surrounding circumstances, they were then visiting the chamber of an insane occupant, afflicted, most likely, with incurable mental alienation.

Of course, amongst the numerous lunatics distributed throughout the cottages in which such persons are lodged, many appeared as infirm of body as they were weak in mind. Several seemed very old, or quite imbecile, and utterly helpless. Some were dwarfs—males as also females; and one female cretin was recognised, although, on inquiry, I understood that similar examples of mental and physical degeneration have been lately of rare occurrence. All clothing is supplied by or under the administration's superintendence; and being generally of good quality, without adopting any uniform mode of dress, it became frequently very difficult to ascertain the demented member from among the sons, daughters, and even master or mistress of the household, where such parties resided. When talking to different individuals composing such family circles, I often could not distinguish one from the other, and, therefore, had to ask my medical cicerone, who always most kindly answered every inquiry, "which was the patient, or were any we then saw actually insane?" Considering the truly small allowance received for board and lodging of lunatic inmates living in such dwellings, their accommodation was good, beds clean, and other appurtenances seemed better than various sane members of these families themselves frequently enjoyed. How the householder could make both ends meet—to use a common phrase—without ultimate loss, appeared very problematical in many instances, considering the limited allowances often received by such parties.

To describe every object seen, during these rural perambulations, would prove both tiresome and superfluous; nevertheless, a few sketches as specimens of the scenes noticed, and patients then visited, may not appear altogether uninteresting. Thus, in an enclosed garden adjoining the cottage of the mad patient in question, an imaginary emperor—a little man—was vociferating and bawling to troops he then believed himself passing under review; ordering Marshal A. and General B., &c., to manœuvre according to imperial command. Our approach did not much disturb the phantoms of this visionary potentate, who merely then followed his daily amusement. In the kitchen of another hamlet,a schoolmaster—at one time the sage teacher of others—was now busily but silently engaged with superintending the broth-pot, in which the family's dinner was preparing. This man had never spoken a single word for years; but being harmless, thus made himself useful. In a third cottage we found a male lunatic, —said to be sometimes excited—zealously nursing one child on his knee, and rocking a cradle, in which another lay asleep; while the mistress superintended her own household duties. Lastly, in a fourth, I could not avoid observing an old man peeling potatoes with a large knife in his hands, but whose arms were strapped by a leathern thong round his back, so as to prevent any lateral movement, while both legs were restrained by hobbles. This afflicted and often very dangerous maniac was still, in some degree, a free agent, to rise up when he liked, to walk out—slowly certainly, discontinue working, or, if it was more agreeable, saunter in the garden adjoining; but being restrained as now described, he could do no injury to others or himself. At least, such was the explanation then proffered to my inquiries.

In the more distant cottages, noisy and excitable patients are commonly placed, as mentioned previously. There, any disturbance or menaces have scarcely an echo. The lunatic meets no person; and living thus away from all neighbours, cannot create much annoyance. Being likewise free to wander—within a certain extent—amongst broom or heather, or over the solitary heath inhabited, a maniac thus often becomes tranquil, it is said, from having nothing but silence or solitude around, and while meeting thereby no opposition.

Within Gheel itself, the better class of patients, paying the highest board, and generally those who are tranquil, or least excitable, for the most part lodge. We visited numerous insane residents, during varied urban perambulations, and were everywhere most courteously received. In several houses which belonged to respectable citizens, the lunatic inmate often seemed like one of the family, if not, indeed, the member most favoured; as well from occupying the best bed-room, as appearing also treated with marked kindness. If frequently difficult, when visiting the abodes of peasants, to know which person was the insane patient, it sometimes became even less easy to ascertain a similar fact, in various *bourgeois* residences. Several lunatics, well-connected, live with hosts occupying even the best dwellings in Gheel; and some inmates called upon seemed very comfortable. Others, again, belonged to the second and third class of patients; in which cases the hosts were usually either shop-keepers, tradesmen, or handicraft persons. Of course, their accommodation and general treatment then resembled, to a certain extent, that in villages; the allowances being analogous.

During these peregrinations, various interesting cases were met with. Amongst others, a most lamentable illustration of insanity attacking an entire family, for one generation, deserves being specially recorded; both on account of its rarity, and otherwise disastrous features. In this instance, four sons and two daughters were all insane; no other children being alive by the same parents. I visited two of these young men, and tall, fine-looking gentlemanly fellows they both appeared. Their brothers we did not see, nor the two sisters, who were then living elsewhere; but being, like the rest, equally deprived of reason. When speaking to these unfortunate insane youths (boarded in two separate dwellings) I was much struck with the calamitous condition to which this stricken family was now reduced; particularly as both father and mother were still living, and could have no prospect whatever of witnessing any change, but for the worse, befalling their entire progeny. As these parties were favourably situated, in regard to worldly wealth, with its oft much-prized enjoyments, and understanding no trace of insanity had ever shown itself on the side of either parent, the sad cases now quoted were therefore highly interesting; being also, in various respects, most remarkable. Parental bereavements like the above, consequently, become even much more deplorable, and deserve commiseration.

When walking about the town, I met several maniacs in the streets; some were enjoying a promenade, others seemed as if returning from their ordinary work, perhaps going homewards, executing a commission for their employer, or otherwise employed. Such parties I only knew were patients from saluting the inspecting physician, or on being pointed out by that gentleman. One young man we encountered may be here specially mentioned. He was carrying an infant in his arms (according to the reply made to our question) home to its parents, in an adjoining street. This statement proved correct, as we subsequently met him at his own lodgings, after having safely deposited the juvenile burden just named with its confiding parents, devoid of any harm or detriment. The great fondness which is frequently exhibited, especially by male lunatics, towards infants and children, forms a marked feature of their conduct at Gheel. They very often take charge of a child in the absence of its parents; will romp with, or be amused by children's prattle; and, if excited, it is no unfrequent result to see tranquillity restored on the approach of such young friends. Sometimes a child will be sent to induce a refractory inmate to return home, when at work in the fields; and these messengers even succeed where older persons fail, or encounter difficulties. Various apt cases of this influence might be detailed, which are neither uninstructive, nor superfluous.

The attachments which frequently take place betwixt parties having charge of lunatics, and the inmate committed to their care, are both interesting and remarkable. Numerous illustrations of this feature in the Gheelois character could be mentioned; but the following examples will suffice, which came under my own observation, or were communicated. When visiting one of the houses in town, where two lunatics had lived during many years with the same hostess, a scene took place which deserves record, both as a genuine outburst of nature, as also highly creditable to one of the party concerned; although similar instances of good feeling are by no means uncommon. After seeing the inmates and their rooms, we then had some conversation with the mistress of this household, who now intimated to the inspecting physician that she had determined to retire wholly from business—viz., keeping a shop and boarding lunatics,—in order to pass the remainder of her declining years in quietness, since she had saved sufficient means for her support, and was now getting both aged and infirm. "But," said the worthy old lady—full of emotion, and actually bursting into tears, "I am heartbroken at the thought of parting with my two poor afflicted friends," pointing to her boarders, "and know not how I can bear our separation. We have lived so long together, that to part is most painful, and I almost feel it will be impossible." This was true human kindness of the highest order, and deserving sincere respect; indeed, such amiable conduct ought to be held up before a selfish world, as an example for imitation.

In many instances, the host and hostess get so attached to their mad inmates, that they grieve when removed elsewhere, even on recovery; but especially, if inexorable death steps forward to seize his prey. Two illustrations of such attachments recently occurred, which well deserve being now related, although other equally interesting examples might be added; but the following are sufficient. In one of these cases, the hostess had for fifteen years carefully tended an inmate, whose friends always paid 300 francs annually. Having become reduced in circumstances, they resolved only to allow 250 for the future. Dr. —— then proposed to transfer this lunatic to a cheaper house, and substitute another paying 300 francs instead. That proposition the party peremptorily refused, with the reply that, "being so much attached to her old, but afflicted patient, rather than make any change, she would keep the poor maniac for nothing." In another case, the hostess had four boarders, one of whom belonged to the lowest paying division, the others to a higher class. Being an excellent person and well-known for great humanity and attention, the relatives of one of the patients—then under her care—wished to place another member of their family in the same house; also offering 200 florins annually. Having a licence to admit only four inmates, Dr. —— proposed to remove the lowest paying patient elsewhere, and so enable Mrs. —— to take the new and higher remunerating boarder. Notwithstanding the promise of double pay, she would not part with her old patient, who had lived in the house many years, and absolutely refused to adopt the suggested arrangement. My informant was so much gratified with this honest and humane person, that the Administrative Committee, on his representation, granted her a special licence to take a fifth patient, as she had otherwise adequate accommodation.

Similar traits of character are by no means rare amongst the inhabitants of Campine; and observers have often remarked, when speaking of this district, that the natives are generally of humane dispositions, kind to each other, and industrious. They are especially conversant with insanity, and much attached to those who are the victims of that calamity. Having, also, been born, educated, and reared, nay, even passed their whole existence amongst lunatics—like the members of many previous generations dwelling in this commune—these peculiar features seem as if second nature, and may serve to explain what otherwise might appear incomprehensible. The Campinois people belong to the Flemish race, and are descended originally from the Normans and ancient Teutons. Hence, the present residents possess in some degree the qualities, bodily and mental, of both peoples. The men do not look as if apathetic or taciturn, but are often keen-minded and also of quick apprehension; whilst the women, besides possessing an inborn aptitude for managing lunatics, are considered generally good-humoured. Having clear complexions, with healthy, well-formed physical conformations, they are said consequently to enjoy much repute as excellent wet-nurses throughout Belgium.

As might be inferred from previous remarks, it is chiefly upon the female members of a family that responsibility rests in managing the lunatic inmates committed to their superintendence. The male members of a household seldom interfere; unless where disturbances occur, or the insane lodger becomes so excited as to require the aid of physical strength, to restrain any outbreak, and so prevent dangerous consequences from ensuing. In our visitations, both in town and to country cottages, it was almost invariably to the female branches of a family—whether wife or daughter—that any inquiries respecting patients were addressed. These seemed always the presiding powers; while their subordinate instruments were frequently children or infants; the male members being usually passive agents, excepting when called on as assistants, upon an emergency. To describe their chief principles of action in a few words, one might briefly say, the system here pursued is based upon *mildness and force*. The first being personified and carried out by the weaker sex; the other through their husbands, sons, and brothers; where that proceeding becomes necessary.

Notwithstanding these and former statements, readers may perceive that personal coercion is still employed in the treatment of lunatics resident at this colony. Even by the rules, a host is permitted to apply the strait-waistcoat, leathern girdles, hobbles, or strong trousers; and to use other kinds of physical restraint, "which have been approved by the inspecting physician, when such means are urgently required." But in all these cases, immediate information must be transmitted to the sectional physician, and receive his sanction; who forthwith makes a report of every circumstance which had occurred to his superior, the inspecting physician. However, should any patient have suffered personal violence, or the unnecessary application of bodily restraint be ever employed, the offending party will not only then lose his or her licence, but may be also prosecuted for damages, and punished for such transgressions.

Compared, certainly, with former years, nay, even subsequent to the visit of Esquirol—when it was no uncommon spectacle to see patients wearing iron chains, walking about in the vicinity of villages, or in the lanes and streets of Gheel—all physical restraint, besides being now of a much slighter description, seems, likewise, far seldomer employed. If used, it is chiefly of the arms, in order to prevent personal violence upon others; although sometimes the lower limbs likewise, lest excited maniacs might escape into the fields, run recklessly through the town, or even disappear altogether. As an ordinary remedy, restraint is strongly condemned by the medical authorities, and ought only to be applied exceptionally. In illustration of this important question, and to show the exact extent of personal coercion actually in force, when I inspected the colony, it should be stated that, amongst the total 774 lunatics then resident, twenty could neither walk fast nor take long steps, in consequence of hobbles on their ankles; five had both legs and arms tied more or less loosely, most of these cases being male patients; whilst eight individuals, chiefly females, wore a camisole, because they were clothes-destroyers and erotomaniacs. Thus making altogether 33 examples; or one case of restraint in nearly every 24 patients. Most of the above lunatics, however, were either sauntering about cottage-doors, or walking slowly in the roads and lanes. Again, others seemed sitting quietly within, but doing nothing; while a few were occupied in household employments, notwithstanding their being then under bodily restraint. Disapproving of physical appliances, it must still be admitted that, here the application of such means is virtually very different in effect from the treatment often pursued in continental asylums.

Although restraint is not yet wholly discontinued at Gheel—as proved by the previous faithful report of its extent and form, observed during my recent sojourn—it cannot be denied but some inconvenience, and even occasionally lamentable, if not fatal consequences have occurred, through the great personal liberty which most patients usually enjoy, while residents of this commune. Quarrels sometimes take place in the streets; patients get excited by incidental causes, that cannot but occur where so much individual freedom everywhere prevails amongst the lunatic inhabitants. The sexes are not so very rigidly kept apart, as at ordinary institutions; whilst, in a district where nearly every tenth inhabitant is really insane, it must be expected almost as a natural consequence of their peculiar position, and mode of living, that agitated and dangerous persons should be either most strictly watched, placed in seclusion, or otherwise secured against doing harm to others and themselves; whereof a terrible instance may be here quoted, which actually happened at no distant period. A male lunatic, having taken offence at being reproved by the then Burgomaster, because he received money from poor people, by pretending to cure various diseases, and for otherwise misconducting himself, threatened that chief magistrate with personal violence, and also said publicly, he would be revenged. The Burgomaster, unfortunately, disregarded these menaces, and therefore adopted no precautions to protect himself against harm. However, one forenoon, when he was passing along the public street, this furious maniac darted out of an adjoining passage, and suddenly stabbed his victim in the back with a knife; whereby M—— got mortally wounded, and died soon afterwards.

Happily, similar instances of violence occur very rarely; whilst illustrations of quite an opposite description might be easily enumerated. But one case will be sufficient on the present occasion, which shows the benefits of removing restraint, where it had been apparently used from dire necessity. Some months ago, an excited patient was brought to Gheel, tightly bound down with ropes to a hand-barrow, upon which he then lay, more like a sack of corn than a human creature; being accompanied, besides the porters, by two men as guards, to ensure safety, while others had been required to secure him, in the manner previously described. Very soon after arrival, this individual was freed from all bodily ligatures, and placed with a peasant experienced in the management of violent maniacs. The lunatic, at first so outrageous, quickly became tranquil, through kind treatment received from his host and hostess. He then began to assist the family in domestic duties, and afterwards went about the garden; till ultimately, his docility and obedience to orders became so marked, that hopes of recovery now seemed well-founded. When passing near the abode of this but recently frantic, and most dangerous patient, we met him tranquilly walking along the road, led by the hand of his hostess's young child, who had been purposely sent by her parent to a neighbouring field, where he was at work, in order to conduct him safely homewards; which the little messenger accomplished successfully.

Struck with the above instance, as also several analogous cases, where young children took charge of lunatics, I made especial inquiry to ascertain whether accidents did not sometimes ensue, from placing such confidence—as similar proceedings implied—in the tranquil conduct of insane persons, should they get excited from slight causes, or unexpectedly. In reply to these questions, I was informed that disastrous results very rarely supervened. Frequently the reverse occurred; since most lunatics are very fond of being in children's society, whilst their talk often proved tranquillizing, and seemed really even to contribute, if not towards their recovery, at least materially to assist in rendering them more manageable; judging from appearances.

The chief feature characterising this colony being that of almost universal personal freedom, in so far as locomotion is concerned, and as the lunatics generally pass much of their time in the open air, or out-door employments; besides, seeing no dungeon walls or even fences exist to curtail their promenades, while going about the country in bye-ways and roads, like ordinary labourers, it therefore cannot appear singular, if attempts be made to escape; nay, are occasionally successful. Nevertheless, only *eleven* instances of that kind occurred throughout twelve months ending the 1st of last September, according to a register kept by the secretary, M. Yerelst, and of which he kindly gave me an official extract. As the expenses of recapture fall upon the housekeeper where the fugitive resided, and who farther receives no allowance during such absence, it becomes the interest of every person to prevent patients escaping; while the local police, besides the gendarmerie of neighbouring communes, being always on the out-look respecting runaway lunatics—for which duty they are rewarded—actual escapes hence become very difficult of accomplishment, as shown by the return above quoted. Indeed, considering the great personal liberty enjoyed by so many lunatics as 938—the entire number under treatment at Gheel throughout last year—it cannot seem surprising should escapes supervene. On the contrary, the fact of so few as only Eleven instances having actually taken place during twelve months, appears even less than might be expected under ordinary existing circumstances.

Upon this point it seems of importance to refer to similar occurrences at other establishments; as, for instance, to the Maréville Asylum, which contained, at the period of my visit, some years ago, 876 lunatics, and, therefore, having nearly the same insane population as that of Gheel. Notwithstanding the *surveillance* exercised at this institution, and although it is enclosed within walls or fences, *nineteen patients escaped* during the year embraced in the Report I published respecting Maréville in No. XVIII. of the *Psychological Journal.* Analogous facts might also be mentioned in reference to other institutions; and therefore, instead of being considered as any objection to Gheel that patients may run away, the proportion even appears less than in some modern asylums, with all their appliances to prevent such contingencies. Rather, the wonder is, truly, that not more than eleven lunatic patients did then take advantage of the liberty they enjoy, and make their escape from Gheel.

Regarding the general movement of insane residents under treatment, during the period referred to in a preceding paragraph, namely, for one year up to the 1st of last September, the new entries of patients amounted to 137; the number cured were only 29; those transferred either to other asylums, or removed by friends, prior to convalescence, were 50 altogether; whilst 74 died. The ratio of deaths hence exceeded, by more than double that of recoveries; which latter, if calculated according to the number admitted, consequently gives about 22 per cent. of actual cures. This proportion certainly appears very small; but considering the numerous incurable cases, their long continuance, feeble physical frames of many inmates, often advanced age, and lastly, the utter hopelessness of ever effecting much, or even any good, in a very large number, may account for such unsatisfactory results, as also partially explain the large amount of fatal terminations. However, respecting recently recorded mortuary details, it may be briefly remarked, that the most frequent apparent causes of death reported, were apoplexy, phthisis, epilepsy, and general paralysis; although many finally succumbed, in all appearance, from old age and exhaustion; or, according to popular phraseology, "*vieillesse et marasme.*"

The large number of fatal cases, now enumerated, do not constitute an unusual amount of mortality, when contrasted with returns of previous years. Thus, amongst the lunatics belonging to the hospice administration of Brussels, which averaged during 1849, usually under 350, and of whom 72 were new admissions, the cures did not exceed 58 cases; while 32 deaths were reported. In 1850, with an average population ranging about 345, and of whom only 46 were recent entries, not more than 17 recovered; whereas 25 died. Amongst which category, it deserves being stated that 10 of the deaths were of old men who constantly had resided in Gheel since 1803, or 47 years at least, whilst one patriarch was on the verge of becoming a centenarian. Such facts seem conclusive evidence, not only of the care bestowed upon lunatic inmates at that colony, but likewise, of the advanced age which they occasionally attain. Further, during 1851, which showed 55 new admissions, and an average of 325 resident patients, 30 died; while only 9 are reported to have recovered. These figures are now quoted to prove that the casualties observed during the past twelve months greatly resembled those met with in other seasons. But better comparisons may be made with the actual movements of patients, recorded during two of the above specified years, throughout the entire colony. For example, in 1849, with a total population of 980 lunatics, the new admissions were 164, the numbers cured 75, the deaths 82, and 11 escapes. Again, during 1850, when the aggregate numbers had fallen to 931, the new cases were 152, cures only 38, and 67 deaths, whilst 10 escaped. The above statistical data—all taken from authentic sources—consequently show that, at this colony, last year's experience varied very little from previous similar periods.

Among the numerous insane residents at Gheel, besides workmen and artists of various kinds, painters, professors of languages, also of music, schoolmasters, governesses, literary persons, and so forth, may be frequently recognised. An interesting example of that kind occurred many years ago, which deserves mention—viz., that of a celebrated violinist, who then became an inmate. This gentleman, having still great love for his art, established musical meetings to amuse the patients; he being leader. That step led to the erection of a large hall—yet existing, in which the founder's portrait now occupies a prominent position. Ever since, similar reunions have continued to assemble in this building, which give much pleasure both to spectators and performers, of whom a proportion are patients; while many also belong to the middle and upper classes of the general population.

Instances have occurred where patients, after they had entirely recovered, felt so comfortable with the parties under whose roof they were placed, and with their abode, that instead of returning home, preferred remaining as boarders with those new friends, from whom they had experienced much kindness and attention when insane. Many such examples might be detailed, but one is sufficient. An insane female, after being some years resident with a *nourricier* at Gheel, became convalescent. She, however, would not depart, but remained permanently. When asked the reason of her unwillingness to leave, replied naively, "I am accustomed to this worthy family, and feel happy. I have known these two daughters of my kind friend here, ever since they were born. I love them like my own children. I am poor, and have no relations; therefore where could I go and be so very comfortable as here? So move I wont!" This was said to the narrator of the above anecdote, after the aged speaker had resided twenty years in the colony.

Although, as a rule, lunatic inmates are not allowed to visit any *cabaret*, tranquil patients may have that permission, at proper hours. Hence, it is not uncommon to see insane guests sitting at the tables of such places of entertainment, either smoking their pipes, amusing themselves with playing cards, or engaged at billiards and dominoes, all the time sipping their beer; while others again are talking together, like any ordinary frequenter of coffee-houses, and from whom they are scarcely distinguishable; at least by casual observers, who, similar to myself, only looked into these apartments out of curiosity, when passing their thresholds.

Acknowledging, without reservation, various peculiar advantages possessed by Gheel, as an establishment for the reception and care of insane persons, embracing certain categories; nevertheless, if compared with some modern asylums, in several features it seems defective. Although not surrounded by high walls, having no securely-locked wards, with often confined and over-crowded dormitories, in which numerous inmates seldom breathe an uncontaminated atmosphere, and then rarely, if ever, associate with sane fellow-creatures, but almost continually dwell in the often baneful society of other lunatics—whereby they become wholly deprived of many social enjoyments, which frequently prove alike advantageous during the treatment of mental, as in physical maladies—the insane colony now under review requires, nay, imperiously demands, various important ameliorations for the relief of suffering humanity. Of these, the most essential and urgent, at present, is an infirmary, adequately large and sufficiently commodious, for its existing numerous lunatic population. This great desideratum has long been acknowledged. But notwithstanding the official reports recently made thereon, and although the subject has been very often discussed, while authorities have strongly recommended the establishment of so necessary an appendage to every asylum, as the one in question, nothing by any means sufficient has yet been accomplished towards remedying the present great deficiency above specified.

A small house or cottage has, no doubt, been appropriated for receiving new admissions, previous to placing such patients in any permanent abode, and where one or two sick persons, on an emergency, might be treated when labouring under physical disease, instead of remaining at their ordinary domicile. Still, the accommodation supplied is altogether inadequate for that purpose ; but especially for observing recently arrived lunatics, so as correctly to ascertain the specific character of their mental disease, previous to locating them permanently elsewhere. This objection being even more applicable, when attacked by acute cases requiring much personal attention, and constant medical superintendence.

An infirmary containing from fifty to sixty beds would prove sufficiently ample for every requirement. The locality selected ought to be central, salubrious, and in an elevated, open situation. The vicinity of St. Dympna's church has been proposed ; and, certainly, this position possesses several recommendations. Wherever the new infirmary may be ultimately erected, 50,000 francs would be judiciously expended on such a building, and could not but confer great benefits on the lunatic population of Gheel ; irrespective of important collateral advantages to its general population, by thus holding out an additional means for properly treating those who might be sent here as patients. To accommodate 800 lunatics—the average ordinary number at this colony—several millions of francs would be required to construct two asylums, even of an ordinary description ; while a much larger expenditure must be incurred, were these structures made palatial, or like some which oftener seem rather built for external show, and to attract outward admiration, than invariably for their interior convenience and useful accommodation.

Neither Government nor provincial ratepayers have ever expended any large sum, on purpose to procure house-room for the numerous lunatic population resident at Gheel. The annual outlay required from different communes is exclusively paid on account of the care, keeping, and maintenance of their indigent insane; not to reimburse any expenditure on buildings. Indeed, the allowance, which frequently averages about £10 per annum—every item included—being less than that often paid at ordinary asylums, even in Belgium, for pauper lunatics, and where thousands of francs have been spent on buildings, there would be true economy in the proceeding advised. Hence, it proves much more advantageous, pecuniarily considered, to place insane patients at Gheel; seeing neither the communes nor public treasury are required to make any considerable outlay, as a commencement. Whereas, such results almost invariably happen elsewhere, in reference to similar institutions. Viewed, therefore, even as a money question, besides under various other aspects, Government should not hesitate to build an infirmary, and also to institute other improvements now admitted as absolutely necessary. Much has certainly been accomplished of late years, to ameliorate the condition of resident lunatics. The new system of management adopted has already produced beneficial effects; and although local authorities may thereby have been curtailed in their former powers, especially with reference to patronage; whilst individual jobbers can no longer successfully pursue their profitable vocation as heretofore; still, many helpless patients have derived important advantages, through several judicious changes already established.

When impartial observers reflect that about 250,000 francs are annually paid to upwards of 450 different householders, for the maintenance of nearly 800 insane boarders, distributed amongst the various families just enumerated, it will be at once perceived, of how much importance such an expenditure becomes to the entire community. In truth, the large sum so received constitutes their chief revenue; quite as much as cotton-spinning in Manchester, or working in metals at Birmingham. Take away their lunatics, and the Gheelois commune would be utterly ruined; unless some new employment or industry were substituted.

In a former page it was stated that Gheel, and its immediate precincts, formed almost an oasis in the surrounding desert. Indubitably, the continuous residence of many hundred lunatics, during nearly twelve centuries, has essentially contributed towards rendering an arid sandy soil—naturally unproductive—into fertile fields and fruitful gardens, notwithstanding its often cold or ungenial climate. Well may modern authorities and natives celebrate the fête of St. Dympna; for truly to that saint originally belongs the long-continued fame of this locality, and also the marked improvements in its soil just mentioned. While again, to such named influences are mainly owing whatever advantages it now possesses over adjacent communes. Hence, much material wealth has been diffused, not only amongst past generations, but residents of more recent times. Had analogous establishments been placed in a sterile highland moor, which originally produced nothing but heather and grouse, or on any Sussex down, where hitherto sheep-grazing seemed its only profitable destination, assuredly both places would have also become, through the continued labour of lunatics, equally productive with this district of Campine. Were such experiments tried, of course many years, perhaps several consecutive centuries must elapse, before similar results could be reasonably expected. Nevertheless, the conclusive exemplification, described in these notes, amply shows what may be accomplished, through the continued physical efforts of numerous individuals congregated together; notwithstanding they may be all victims of mental alienation.

One great and truly peculiar feature, which has, during now many centuries, always characterized this singular colony, ought never to be forgotten—namely, when lunatics, everywhere else, were too often treated more like so many wild animals than as human beings, and even frequently chained, or lying on straw in dark noisome dungeons, here, the sufferers from similar mental maladies breathed an open, more healthy atmosphere, lived generally quite free, like other members of the family where they resided, and in whose occupations, enjoyments, or even annoyances, they often participated as ordinary inmates. Indeed, many never suffered from any bodily coercion, unless under certain circumstances. In fact, *No restraint* appeared the great maxim at Gheel; physical confinement being the exception. Whereas, throughout the civilized world generally, restraint—frequently of the most severe description—was formerly almost the invariable rule, bodily freedom proving then of exceedingly rare occurrence.

But however remarkable this ancient rural refuge, for demented members of frail human nature, may appear, and although in operation now during many hundred years, the locality and its famed attributes were always, and even now still continue, very imperfectly known, not only to an ignorant public, but even by the medical profession, who, generally speaking, remained, unless to a very limited extent, scarcely cognizant of Gheel's actual existence. Recently, the colony has become somewhat better understood. Again, since various reforms have already been effected, besides others now in progress or proposed; while the facility of travelling thitherward is at present reduced to a forenoon's journey from Brussels, with the certainty of obtaining every personal comfort visitors could desire, when residing in Gheel; doubtless foreign physicians will very soon become as familiar with *Kempen Land*, and its peculiar features, as they are now in regard to most European districts and their peculiar institutions. Whether such extended knowledge may lead to the establishment of similar insane colonies in neighbouring countries, at present seems problematical; although many strong arguments might be easily advanced in support of that proposition. This much certainly may be said, no asylum for lunatics throughout the universe can boast of having had such a lengthened experience as the one in question. That assumption, at least, I firmly believe, cannot be disproved, with any show of reason.

Notwithstanding the prestige of great antiquity; the acknowledged physical benefits so frequently produced; the freedom which now most patients enjoy—even greater than ever; the almost general adoption of "*no restraint*," and many other advantages—to say nothing of recent ameliorations effected, or seriously contemplated—an opinion exists in some influential quarters that, instead of still further improving the colony of Gheel, it ought to be entirely broken up and discontinued. In short, the insane inmates should henceforward be consigned to public asylums, shut up within four stone walls, only associate together, and never be allowed to enjoy the society of their own fellow-men, until they also have become victims of mental derangement. No greater and more disastrous mistake could be committed, than to carry out such an absurd proposition. Correct abuses; still further improve internal discipline; remunerate the medical staff more liberally; make the communes pay higher allowances for their pauper patients, so as to ensure increased comforts to such sufferers; supply additional means towards promoting trades and manual employments amongst men, as also work for women; and lastly, but still not the least essential, forthwith establish an infirmary both for the temporary reception of acute cases, and of those inmates who may become attacked by physical disease. Doing these things effectually, in my opinion, would be the most proper course to pursue—not, certainly, by adopting any thoughtless scheme of destruction.

Instead of committing such an act of sheer Vandalism, insane rural colonies should rather be established elsewhere, and thereby take advantage of former practical knowledge—based upon the long experience thus obtained. Other countries might even advantageously imitate the example here furnished—which appears, for many reasons, truly philanthropic—in place of always erecting immense prison-looking buildings, or large, magnificent, palatial residences, for pauper lunatics, which are wholly at variance with all their previous habits, lives, or associations. Besides these reasons, asylums become not only very expensive, both in construction and management, but frequently prove not the best adapted for protecting, treating, or occupying judiciously that class of the population.

Nowhere in Europe is any analogous establishment to be found—at least, within my knowledge. At Zaragossa, in Spain, an insane colony was said to have formerly existed; but to what extent, or if it still receives any inmates, I am unable to procure certain information. However, Iberti, an author of repute, when mentioning this institution in 1791, says—"Here, lunatics have been employed, long prior to the present period, in daily labour, either within the hospital, or adjacent fields." Afterwards, the same authority adds, "that rich patients who did not engage in manual occupations rarely recovered." These quotations, therefore, become both conclusive and interesting. Again, in the north of Scotland also, about the middle of last century, a farmer then obtained, it is said, considerable celebrity by the treatment successfully pursued with insane persons, whom he took as servants and labourers, to cultivate his farm, or to pursue various out-door manual employments. The place was, in fact, another Gheel, although upon a very limited scale. How long this small lunatic colony continued in operation, or with reference to its particular features, even rumour is now silent; and the fact of such a place having once existed seems now almost, if not wholly, forgotten.

Indubitably, the principle then acted upon continues recognised, as incontestably shown in many recently-erected county lunatic asylums, where large farms and gardens are frequently now attached, so as thereby to occupy patients in open-air labour, rather than confine them in close wards, waiting-rooms, and workshops; or even placing such parties in walled court-yards, however spacious. Virtually, therefore, a fictitious or rather miniature Gheel is often now appended to various public institutions for the insane, but without possessing the peculiar advantages characterizing that unique establishment—viz., domestic association with sane persons, as likewise, the demented living usually at large, among ordinary workpeople. Irrespective of several drawbacks, these agricultural appliances are nevertheless most beneficial. Consequently, their operation should be greatly extended, so as to become, in a higher degree, both useful and sanative to numerous inmates.

In addition to the examples above quoted, an important application of a similar system at the Devon County Asylum, during the past year, deserves being mentioned. According to the Report of that institution for 1856—drawn up by its able medical superintendent, Dr. Bucknill—"a limited number of patients have been discharged on trial, and boarded with neighbouring cottagers selected as trustworthy and suitable persons. In several instances the women of these cottages have acquired some experience in the right management of the insane. Some of them have been employed as occasional attendants in the wards of this asylum; and others, having been attendants or domestics in the asylum, have married asylum artisans, or other persons living near. This experience has made them willing to accept, and qualified to undertake, the charge of such inmates of their houses. Both the patients and the persons having charge of them feel themselves under the eye of the medical superintendent, who visits them unexpectedly. The plan promises to work well. The patients are happy, and extremely well satisfied with the arrangement." In a recent letter with which I was favoured from my friend Dr. Bucknill, that gentleman further says:—"The cottage treatment of a few selected patients mentioned in my last Report has fully answered my expectations. The patients are contented and happy, and bear willing testimony to the kindness and consideration of their hosts." This forms a good commencement, and, if carried out further, will doubtless prove equally satisfactory. Considering that the average primary outlay, of most public lunatic asylums, ranges about £200 per patient, an annual expense of nearly £10 is thus incurred, before any inmate can be fed, clothed, or treated. Hence, it appears the buildings in which the pauper insane of England are placed, alone cost the country quite as much as lunatics of the same class in Belgium usually do for lodging, support, and every requisite. These are important considerations, and well deserve the mature examination not only of ratepayers, but philanthropists. At the same time, seeing a successful beginning has been already made at the institution just named, such an excellent example becomes more worthy of imitation elsewhere.

Before taking leave of the ancient and truly interesting establishment, which has formed the subject of previous remarks, two most important questions therewith connected, at least deserve some special observation—namely, does the residence of so many lunatics in this commune, their free intercourse with the general population, and the association of sane and insane persons—ordinarily without restriction, influence the mental condition of the rising generation? Again, do immoralities ever supervene, in consequence of the two sexes being in the habit of often meeting each other, as if actually living in common worldly society? Upon both these topics, no opinion can be more valuable or conclusive, than that of Dr. Parigot, who resided, during several years, as inspecting physician at Gheel, and had thus ample opportunities for making valuable observations.

In reply to the first question—viz., whether the association of insane persons with the general population actually produces any injurious effect upon the mental condition of natives belonging to this commune? Dr. Parigot very obligingly wrote me as follows.

I give his observations in the original French, so as not to weaken their force or significance, by translation :—" Je vous dirais qu'au premier abord, lorsqu'on voit ce qui se passe en général à Gheel, on serait assez tenté de croire les Gheelois, si non fous, au moins assez excentriques. En second lieu, un étranger, s'il s'en rapportait à ce que disent et racontent—1$^{mo}$, les villageois et citadins qui avoisinent Gheel; 2$^{do}$, les membres de l'administration provinciale et ceux du tribunal dont Gheel ressort—il s'en irait convaincu que les Gheelois ont subi l'influence du contact de leurs aliénés ; car toutes les personnes qui ne sont pas de Gheel, moitié par dérision, moitié à cause des faits qui arrivent à leur connoissance, et dont ils ont à juger, les considèrent comme des aliénés ; de là, sobriquet *Gheelsche Zotten.* Toutefois, mon expérience n'est pas d'accord avec ces données, et j'ai remarqué—1$^{mo}$, que Gheel ne fournissait relativement pas plus d'aliénés que les localités voisines ; et 2$^{do}$, que les excentricités apparentes dépendaient de causes amenées plutôt à la suite des aliénés (comme la somme de 250,000 francs qu'ils apportent à Gheel), que par le contact des aliénés. Je m'explique ; vous savez qu'il y a deux espèces de Gheelois—l'habitant de la ville même, et celui de la campagne de Gheel. Le premier a plus ou moins abandonné le travail des champs pour le commerce de détail, ou pour la spéculation, même celle de se charger de *l'entreprise des aliénés.* Le second est resté campagnard, et est devenu avec l'aliéné l'objet de la spéculation du citadin ; aussi, le paysan n'est point en question, quand on parle des Gheelois ; mais le citadin spéculateur et fainéant, grand beuveur de génièvre, est devenu difficile à conduire, assez chicaneur, entêté, et fortement excentrique. On ne peut pas nier non plus que cette grande habitude du *laisser faire* pour les aliénés n'ait contribué à relâcher sa manière de voir les choses, ses conceptions, et que sa volonté n'ait subi une certaine modification, tant sur le contrôle de soi-même que sur les actes ; mais, cependant, tout cela n'est pas de la folie. Au reste, vous concevez, Monsieur, que si le principe de la contagion de la folie était admissible, Gheel, depuis le temps qu'il est soumis à cette influence, ne serait pas un *peu excentrique,* mais, *bien fou à lier*—ce qu'il n'est pas. Au contraire, j'ai toujours admiré, au milieu de bien de défauts, l'immense charité et l'amour souvent désinteressé des habitants envers les aliénés, par le temps qui court en vue de ce que la soif de l'or produit dans le grand monde. Le désinteressement n'est-il pas aussi de la folie ?"

With reference to the second point of inquiry—namely, do immoralities oftener supervene amongst the insane residents of Gheel than elsewhere ? Dr. Parigot likewise observes, in answer to that equally important question—" La vie de famille est la véritable et unique base du réglement de police intérieure de Gheel. C'est sur elle que se modèle la vie des aliénés, et leur moralité en dépend. Dans les familles on ne sépare point les sexes; et cependant, il est infiniment rare, qu'il s'établisse des rapports criminels entre les membres. Les aliénés étant, chez les Gheelois, admis sur le pied d'enfants, qu'il faut *conduire* et *surveiller*, j'attribue à cette surveillance intime, plus qu'à toutes les récommendations de l'administration, de n'avoir trouvé en sept années que quatre cas de grossesse parmi les aliénées; cependant, ces dernières sont en moyenne en nombre de 500. Mais, ce qui ne doit point vous échapper, c'est qu'à Gheel peu d'aliénés sont inoccupés, surtout les femmes valides; et que justement, ceux dont les instincts animaux sont le plus développés, se trouvent dans ce dernier nombre, et seront ceux qui auront le moins d'occasion de s'y livrer. Enfin, le nourricier considère un pareil fait dans sa maison comme un *deshonneur*."

These observations being based upon extensive personal experience, must carry conviction, in reference to the points now mooted; more especially, as Dr. Parigot's conclusions coincide with those enunciated by other authorities, equally competent. Although any remark of mine on such subjects would be valueless, I would nevertheless observe that Gheel seemed a very quiet town, considering its size. Indeed, it was certainly much more tranquil than many other localities I could name—numbering 4000 residents. During daytime, nothing unusual occurred; whilst at night, silence and repose reigned, apparently, everywhere supreme. Nay, an unusual quietude even prevailed when an infantry regiment—about 1000 strong, and returning from the camp at Beverloo—passed through Gheel; where they were billetted with the bourgeois population, during one of the nights of my sojourn there. Greater bustle and confusion would have certainly ensued in most English or Scottish towns, by the marching in one day and out the next, of so large a body of soldiers, headed by their military band, than then prevailed. The absence of any excitement was indeed remarkable. Hence, were I to hazard an opinion, derived during my brief residence in Gheel, it would be that the capital of Campine seemed more orderly, and less noisy, notwithstanding 250 free lunatics dwell therein constantly, than almost any other equally populous place possessing only sane inhabitants, which I had ever previously visited. Such a remark, however strange it may perhaps appear, being really no exaggeration.

Although neither peculiar to this locality, nor uncommon, still as the immediate vicinity of Gheel supplies an instructive illustration of what may be accomplished, in originally an unprofitable soil, by human labour even of lunatics, if applied consecutively to its cultivation, I would specially direct attention to the district in question; seeing, it has thereby been changed from an almost Siberian desert—as various environs even yet remain—into cultivated fields highly productive. Instead, therefore, of constructing new palatial asylums, which is now often the case, in situations where the ground proves valuable in consequence of its fertility, public authorities should profit by the results noticed at Gheel, and henceforward locate all future county institutions for lunatics on moors or waste commons, in place of purchasing, sometimes at an exorbitant price, tracts of ground already in cultivation. Of course, the district chosen should possess ascertained salubrity. This step is too frequently adopted because the position selected seemed beautiful, and might, in consequence of an elegant architectural structure afterwards erected thereon, become the admiration of passing travellers; and would then be pointed out as strong financial evidence of county magistrates' liberality, besides showing the great care they take of their pauper insane.

Intimation having been officially given to the metropolitan county magistracy, respecting the necessity of erecting a third public asylum, that proposition therefore supplies a good opportunity for carrying into effect the principle now enunciated. If such movement be made, few places really seem better adapted for that purpose than Hounslow Heath. Sufficient ground could be there easily obtained, which must doubtless become, were a large population constantly located, much more productive than at present; while it would supply an ample space for out-door physical labour. However, any suggestion now mooted being only shadowed forth for subsequent consideration, I need not further pursue the inquiry. Still, having witnessed very marked beneficial results, which an analogous mode of proceeding has effected in the bleak "Kempen Land" of Belgium, similar consequences would most likely follow, I really believe, were an analogous, or even a partially similar institution established on any barren but healthy heath, within the limits of Middlesex.

### *General Remarks.*

Compared with various public asylums of France, Scotland, or England, many insane establishments of Belgium are very far inferior, in reference to the accommodation they supply. Several old convents—never constructed for the reception of lunatics—having been appropriated for such purposes, easily and at once explains the marked difference which often prevails. Viewed, however, in regard to their condition, prior to the governmental inquiry, not many years ago, great improvements have certainly taken place since they were placed under public inspection.

The most objectionable are suppressed ; and others having undergone important ameliorations, it must be acknowledged, much real good has recently accrued throughout numerous institutions. Take, for illustration, a Report of the Commission of Investigation for 1841, wherein it is stated, "In nine establishments visited, we found iron fetters and chains were used; and in seven others, their employment was suspected; whilst blows and bad treatment, inflicted upon inmates of certain asylums, were too real and too frequent."

Contrasted with this sad statement, reference may be made to the Commissioners' Report for 1853, which observes, "The publicity given to several deplorable facts previously reported has attained the object proposed; various barbarous instruments, which proved the absence of science as of humanity, are now banished to the museums of antiquarians." Again in 1856, the same public authorities report that, "reforms have been introduced in different asylums, with reference to their domestic arrangements; important modifications are made in others affecting the comforts of inmates; various buildings have assumed a more pleasing appearance; wards seem better furnished; greater order prevails; services are performed with more precision, and the surveillance of patients is better understood. In fact, most Belgian asylums have now entered on the path of decided progress." When the new building close to Ghent is completed, the advance must, doubtless, become even more rapid; as then, assuredly, that public establishment for the insane, besides leading to the erection of similar asylums elsewhere, will, moreover, serve as an excellent model for imitation throughout the whole kingdom.

Belgian lunatic institutions are generally distinguished, from those of most European countries, in being very often exceedingly small, and from having usually but few inmates; whereas, the latter are large, and often very populous. Many were originally founded by religious congregations, and even still continue under the management of clerical personages. Some appertain to workhouses and hospitals; whilst in a large majority, all interior services are performed by "frères" and "sœurs" of different religious corporations; scarcely one-third having ordinary, or laical attendants. Again, about half are administered by, or under the authority of various directing Boards of civil hospitals; whereas, only one establishment—namely, the asylum for male lunatics at Froidmont, near Tournay—belongs directly to Government; who have delegated the management to a special commission. At present, this small receptacle is capable of containing from 76 to 80 patients, all being of the male sex. However, as it is proposed to expend upwards of 80,000 francs towards improving and enlarging its accommodation, very soon sufficient space will be supplied for receiving 150 demented inmates within this governmental institution.

Respecting the dietary of insane patients—always so important an item in their treatment—it may be here observed that, the minimum allowance for adults in Belgian asylums should never be less than $32\frac{1}{6}$ ounces of raw meat, $96\frac{1}{2}$ ounces of bread, 13 ounces of butter, with $10\frac{1}{2}$ pints of beer per week. These quantities may, however, be diminished by one-sixth, in the cases of women and young persons under fifteen years of age; whilst the regimen during fast-days, and for sick persons, must be regulated by the authorities of individual establishments, according to special regulations. The above quantities constitute but meagre fare, truly, for grown-up human beings, and scarcely seem sufficient. Nevertheless, the scale is much above what it often was in former years, when competition prevailed amongst different speculating parties; who then farmed out, contracted for, or traded in lunacy. At that period, many lunatics were almost starved, as the feeding of these unfortunate victims was often reduced, by hard-hearted speculators, to the very lowest limit likely to maintain vital existence. Indeed, it is said, some were even fed exclusively upon rye bread and sour milk! During the last two years, the Belgian labouring poor have suffered great privations, in consequence of the scarcity of food: hence, admissions into asylums were much more numerous than previously. Therefore, knowing mental disease originates frequently through want and misery, such contingencies, however common, could be easily explained by the sufferers' insufficient nutriment and physical degeneration.

Small as the amount of animal food just specified may appear, it should be still remembered that the quantity is all consumed during five days of the week; seeing every Friday and Saturday are "*jours maigres*," when a portion of fish or a few mussels are distributed. Should these articles happen to be dear and scarce, then eggs or some equivalent become substituted. Notwithstanding such additions, besides occasionally potatoes, there cannot be any question that too much bread is usually eaten, while the proportion of animalized matter is very inadequate. The regimen in lunatic asylums seems, however, much more nutritious than inmates there confined often previously enjoyed; among whom many, through living almost constantly on vegetable diet, had become debilitated in body, pale and of a yellowish countenance. Hence, while their spirit is thus weighed down, or broken by mental and bodily sufferings, these influences lead to, or facilitate, attacks of insanity in various instances.

That this picture is not overdrawn, might be conclusively proved by numerous yet apt illustrations; although two need only be now quoted, since they appear conclusive. In one of the largest prisons of Belgium—recently containing upwards of 1500 male inmates—a high official authority told me that not more than 300 were fit for military service. This statement seems the more remarkable, seeing most prisoners usually attain improved bodily health after confinement in these well-managed State receptacles. Another friend—a physician of much experience, and formerly in the military service, but now medical inspector of conscripts for a large district—stated also, in proof of the present deteriorated bodily condition, both of the urban and country population in Belgium, that within the last very few years, he had actually rejected upwards of 7000 young men—drawn for military duty, in consequence of their physical incapacity or degeneration; whilst the proportion he admitted as fit ranged much lower than in any previous similar period, according to his experience. These statements deserve serious attention by Government.

Such facts speak volumes; and even casual observers travelling in Belgium, need only make use of their eyes, when visiting large congregations of people assembled in workshops, and various public establishments belonging to towns, or many humble dwellings of the rural populations, to feel convinced respecting the truth of analogous conclusions. Poorness of diet, prevalence of scrofula—often a consequence of the former—with mental sufferings, from misery and bodily privations, always have a marked disastrous influence in producing insanity. Consequently, it cannot be considered surprising, if psychal maladies are said to have become recently more numerous. Towards which result—as admitted in the Commissioners' late Report—an education that developes the passions, and engenders artificial wants, besides commercial or industrial crises, wrecked fortunes, sensual life, as also disordered habits, have all materially contributed; irrespective of even causing other baneful consequences.

Admitting unequivocally the importance of various improvements, lately made in different Belgian institutions, one department still imperiously demands organic changes—namely, the medical staff of these establishments. No public asylum yet possesses a resident physician or surgeon; and in many, the remuneration is utterly inadequate for the important services rendered. Nay, at some the salary is so insignificant, that the office may be almost considered gratuitous, since the annual pay occasionally does not exceed 200 francs! That is most pitiful parsimony. Therefore, it is no wonder if professional attendance, at such institutions, is thus considered of very secondary importance. Indeed, it has been observed, with reference to establishments so constituted, that the patients being thus abandoned to the sole efforts of nature are rarely cured, and thereby often become a permanent charge to their respective communes.

Receiving such inadequate remuneration, there consequently exists very small encouragement for professional men dedicating their time, chiefly, to the study of mental diseases. Hence, very few psychological physicians are found in Belgium ; while the medical officers of many asylums, being ordinary practitioners, visit these receptacles much the same as if attached to ordinary civil hospitals, and often take little or no part in the direction of the institution, properly speaking. At most insane establishments, that duty is usually left to laical or religious directors, by whom the medical attendant is frequently considered as only an appendage, not the chief moving power—which he ought to be invariably—in everything connected with the moral, physical, and medical treatment of lunatics. This truly injurious system requires immediate alteration. A resident physician ought always to be appointed at every large asylum, possessing paramount authority in his own department. He should, besides, be amply remunerated, so as to secure talent and experience. To do otherwise, is very false economy. Therefore, instead of spending money lavishly in bricks and mortar, to make future new buildings assume palatial imposing forms—which cannot so cure madness, more encouragement should be everywhere given to fully qualified medical men, who only should fill such responsible appointments.

Another feature characterizing several institutions, lately visited, merits criticism and requires amendment. I now allude to the too frequent appointment of religious "frères" or "sœurs" in lunatic asylums, and hence the exclusion of lay persons from various employments. For superintendents in particular departments, as teachers, task-masters, and in several other capacities, such parties are generally admirable assistants to the superior authorities; whilst the devotion, especially of sœurs to their frequently irksome duties, is often most exemplary. But having likewise, according to the rules of their respective orders—with which they are classed—to perform many ceremonial and religious offices—both early and late, besides even during night-time—much of their attention is thereby withdrawn from the inmates, upon whom they ought then to have been in attendance. To exemplify the effects of these obligations, a simple fact may be here given, as an illustration. At one institution inspected, where the whole attendants—upwards of twenty—were cassocked "frères," suddenly many we saw occupied with patients disappeared from the court-yards—then perambulated—but whom I afterwards recognised in the chapel at their devotions, amounting to fifteen in number; but wholly without any audience! During the period thus occupied—although not more than twelve or fifteen minutes—very few frères were noticed elsewhere. In reality, the patients seemed left almost entirely alone, and thus to take care of themselves. As similar movements, however, recur from six to eight times during every twenty-four hours, —even throughout the night,—both winter and summer, these duties assume a very serious aspect, with reference to the accumulated time dedicated to such services, which are therefore not employed for the insane residents' immediate benefit, however otherwise to these performers themselves.

Mooting such questions certainly touches tender and debateable ground, especially in reference to Catholic countries and practices. Still, being particularly struck on witnessing similar proceedings, in more than one establishment, and conscientiously believing they entirely concerned the religious brothers so engaged, not the victims of madness under treatment—I am now led to notice the subject, thinking many ceremonies then performed, in whatever way or aspect they may be appreciated by various parties, seemed misplaced in a lunatic asylum, and even subversive of its regular discipline.

Equally strong objections cannot be urged respecting the *sœurs de charité* placed in female institutions. However, even then, the time of such attendants is often too much occupied elsewhere, rather than with patients under their charge. Therefore, it also requires some amendment. Besides which, as both male and female members, of all external religious orders, are affiliated to powers beyond the asylum precincts in which they actually reside, and whose superior behests they must obey implicitly, such an *imperium in imperio* is, I think, by no means desirable. When resident medical officers, possessing dictatorial powers, shall be attached to all public lunatic asylums, and especially those under the absolute control of Government lay commissioners, doubtless the strong objection now specified to the present system will then be materially modified; perhaps altogether removed.

Nevertheless, without discussing any further the above, and one or two minor questions, but upon which hypercritics might animadvert, every impartial person must admit that Belgium has at present zealously entered on the path of progress and improvement. When the new asylum at Ghent is completed, besides other analogous institutions, which must soon follow so good an example, and while men like Guislain and Ducpetiaux direct public opinion, there cannot remain any doubt that the contrast which this country will exhibit in a few years, compared with no very long bygone period, must be altogether different from its former condition, in reference to lunatics. Consequently, it may be fairly anticipated that various ameliorations which have recently been accomplished in France, and throughout the British dominions, will have soon numerous imitators while adopting modern improvements.

Having now attempted to sketch briefly, in previous pages, the chief observations made during my recent visit to the lunatic asylums of Belgium, I must here bring these Notes to a conclusion. Nevertheless, prior to doing so, a most pleasing duty still remains for fulfilment—namely, to offer the narrator's best thanks to numerous gentlemen with whom he came in contact, during his late excursion, for the kindness and uniform civility everywhere experienced. This is really no unmeaning expression. On the contrary, it enunciates what was then deeply felt, and will long be retained in grateful remembrance. To MM. Guislain, Ducpetiaux, Van Hecke, Bulkins, and last, but not least, to Dr. Parigot—now resident in Brussels, his best thanks are especially due, for the pleasure experienced in their society, as likewise the very valuable information he then obtained. Truly, without such advantages, many previous remarks, which now appear in the pages of the *Psychological Journal*, must have otherwise proved both barren and uninteresting; perhaps, never been narrated.

To the Minister of Justice, his Excellency M. Nothomb, I would further presume also to tender very sincere obligations, for his great courtesy in giving me a special letter of introduction, with which I became honoured; whereby, every facility was afforded to visit asylums, prisons, and other public institutions—as that missive stated, " dans tous les détails"—or in reference to any point I might then desire to investigate, and thereby procure authentic information. Furnished with the above authority—a real " Open, Sesame"—and being also supplied with a " Carte de route," most kindly traced by M. Ducpetiaux, who, besides being a Commissioner in Lunacy, is also Inspector-General of Prisons, I thus was most politely received at every establishment; while many doors and documents were readily opened whenever desired.

Notwithstanding the subject may seem, perhaps, rather irrelevant to the questions discussed in previous pages, I would observe, in concluding my narrative, that however inferior most Belgian lunatic asylums may really appear, in various important phases, when contrasted with many throughout France and England, still, in regard to prisons, as also reformatory institutions for juvenile delinquents, or adult criminals, Belgium need not fear any comparison; nay, in several respects, there is even some superiority. Philanthropists, or persons taking an interest in the punishment and reformation of criminals, should therefore visit the " Maisons de Force" at Ghent and Vilvorde; the Penitentiaries of Liege and Charleroi; the reformatory prisons of St. Bernard for males, near Antwerp, and that in Namur for females, since each will amply repay minute inspection. But no institutions, in my opinion, deserve notice more than the Government colony at Berneem, which contained 372 girls on the day of my visit; and especially that also in the vicinity, called Russeylede—having 430 boys and lads under its roof. Each are admirably conducted, and confer much benefit on the young population they contained. Apparently, the latter reformatory even surpassed Mettray, in France, which I have likewise seen, although now some years ago; as also various similar juvenile establishments noticed elsewhere. These two interesting localities being within twelve miles of Bruges, and to which the Ghent Railway conveys passengers more than half the distance, either are, consequently, of easy access. Hence, a journey thither becomes an agreeable promenade, which may be accomplished without difficulty; and assuredly, it will amply repay any trouble, by whomsoever undertaken. Both institutions confer great credit on the Belgian Government, and especially upon King Leopold, who takes an especial interest in their prosperity. Indeed, his Majesty had only recently visited each to see personally how they were managed; when this condescension, of course, gave much satisfaction to every one concerned, and all appeared highly pleased with the royal patronage they so well deserved.

---

